# Supplementary material for: Genetic comparisons of interleukin-17 reveal a framework for complex signaling evolution
Source: bioRxiv. 2026 Apr 14:2026.04.13.718218. Preprint. [Version 1] doi: 10.64898/2026.04.13.718218 (PMC13105070; doi:10.64898/2026.04.13.718218)
Supplement: Supplement 3 [file media-3.zip › Supplemental files for paper/ERC/IL17xIL17_comparisons/IL17F_IL17RE.pdf]

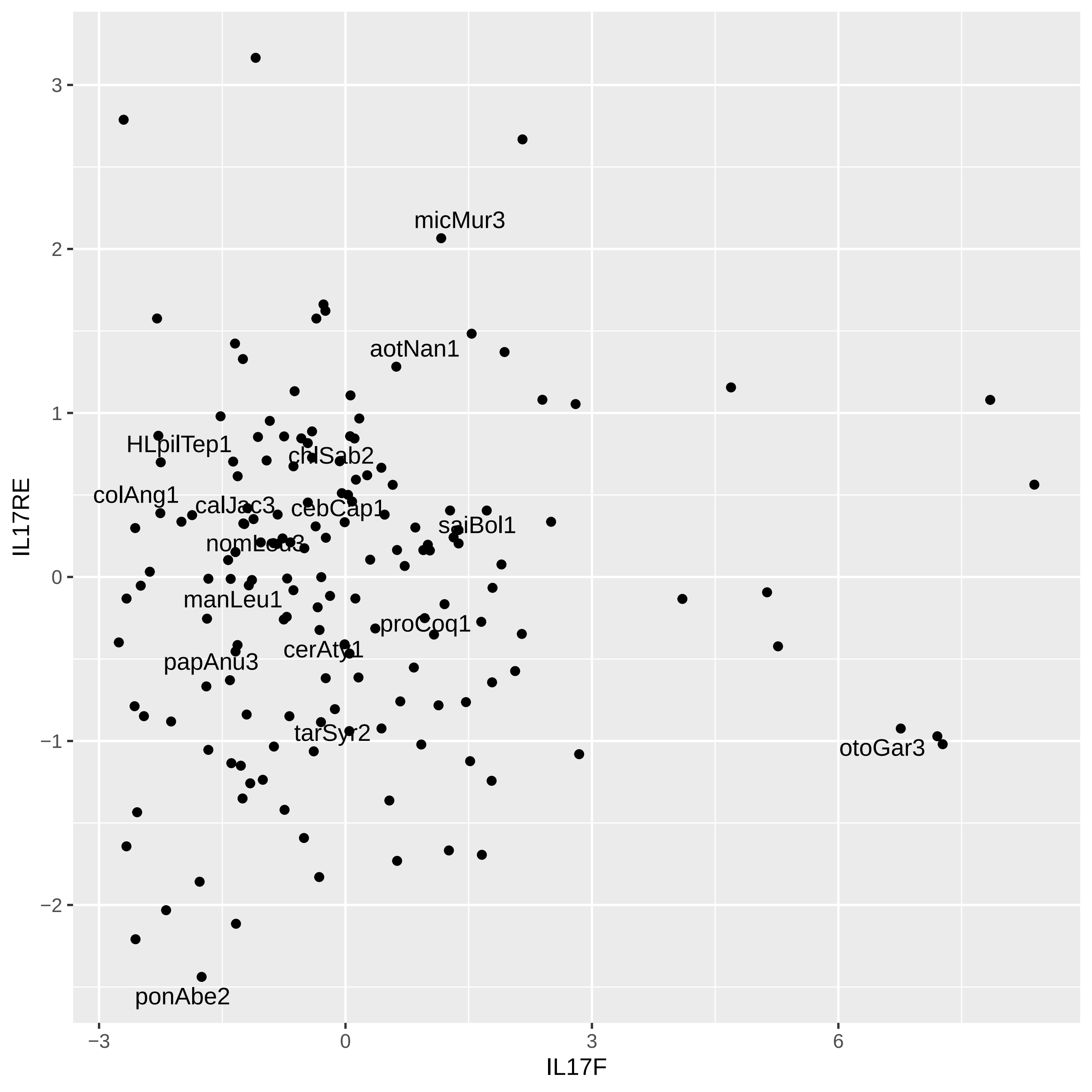

micMur3
aotNan1
HLpilTep1
chlSab2
colAng1
calJac3
cebCap1
saiBol1
nomLeu3
manLeu1
proCoq1
cerAty1
papAnu3
tarSyr2
otoGar3
ponAbe2
IL17RE
IL17F
3
2
1
0
−1
−2
−3
0
3
6
